# Interaction Mediated by the Putative Tip Regions of MdsA and MdsC in the Formation of a *Salmonella*-Specific Tripartite Efflux Pump

**DOI:** 10.1371/journal.pone.0100881

**Published:** 2014-06-24

**Authors:** Saemee Song, Soonhye Hwang, Seunghwa Lee, Nam-Chul Ha, Kangseok Lee

**Affiliations:** 1 Department of Life Science, Chung-Ang University, Seoul, Republic of Korea; 2 Department of Food and Animal Biotechnology, Department of Agricultural Biotechnology, Research Institute for Agriculture and Life Sciences, Seoul National University, Seoul, Republic of Korea; Centre National de la Recherche Scientifique, Aix-Marseille Université, France

## Abstract

To survive in the presence of a wide range of toxic compounds, gram-negative bacteria expel such compounds via tripartite efflux pumps that span both the inner and outer membranes. The *Salmonella*-specific MdsAB pump consists of MdsB, a resistance-nodulation-division (RND)-type inner membrane transporter (IMT) that requires the membrane fusion protein (MFP) MdsA, and an outer membrane protein (OMP; MdsC or TolC) to form a tripartite efflux complex. In this study, we investigated the role of the putative tip regions of MdsA and its OMPs, MdsC and TolC, in the formation of a functional MdsAB-mediated efflux pump. Comparative analysis indicated that although sequence homologies of MdsA and MdsC with other MFPs and OMPs, respectively, are extremely low, key residues in the putative tip regions of these proteins are well conserved. Mutagenesis studies on these conserved sites demonstrated their importance for the physical and functional interactions required to form an MdsAB-mediated pump. Our studies suggest that, despite differences in the primary amino acid sequences and functions of various OMPs and MFPs, interactions mediated by the conserved tip regions of OMP and MFP are required for the formation of functional tripartite efflux pumps in gram-negative bacteria.

## Introduction

*Salmonella enterica* is a facultative intracellular pathogen that infects animals through the oral-fecal route via ingestion of contaminated food or water, and causes gastrointestinal and systemic diseases [1]. *Salmonella enterica* serovar Typhimurium is a well-characterized strain of *S. enterica* that is associated with several symptoms of gastroenteritis, such as vomiting, diarrhea, and abdominal pain in humans. Gastroenteritis is a disease of great concern worldwide [2].

In gram-negative pathogens such as *Escherichia coli* and *S. enterica*, efflux pump systems are employed as resistance mechanisms to promote the active transport of antibiotics and other drugs. Most multidrug efflux pumps consist of three essential components: an inner membrane transporter (IMT), an outer membrane protein (OMP), and a periplasmic membrane fusion protein (MFP) [3]. By using a proton gradient or ATP hydrolysis as an energy source, the IMT expels substrates through the central channel of the OMP, which spans the outer membrane [4]. These two components are joined by the MFP, which is located in the periplasm, to form a functional efflux pump. The crystal structures of several MFPs have been determined, and they are divided into four domains: the membrane proximal domain (MP), the β-barrel domain, the lipoyl domain, and the α-hairpin domain [5]–[9]. AcrAB-TolC is the major multidrug efflux pump in *E. coli* [3], [10]–[12]; AcrB is the RND-type IMT, AcrA is the MFP, and TolC is a homotrimeric OMP with a 140-Å long body that forms a uniform cylinder with a 35-Å internal diameter [13]. TolC consists of three distinct domains: a 12-stranded β-barrel, an α-helical barrel, and a mixed α/β equatorial domain. The α-helical barrel creates the tunnel through the periplasm [13]–[15]. Although each of the structures that contribute to the tripartite efflux pumps has been determined, the modes of assembly and channel opening mechanisms are not yet fully understood.

MdsABC (*mds* stands for *m*ulti*d*rug transporter of *Salmonella*) is a unique multidrug-transporter of *Salmonella* that is not present in *E. coli*. However, these two bacterial species have seven other pumps in common [16]. The RND-type transporter (MdsB), putative MFP (MdsA), and OMP (MdsC) of MdsABC are encoded by a single operon (*STM0351*, *STM0352*, and *STM0350*). Generally, because RND-type drug efflux pumps tend to use TolC as their OMP, which is an OMP found in both bacteria [4], [17]–[18], the operons encoding RND-type drug efflux pumps usually lack OMP genes [18]. However, *mdsC* is located in the same operon as the genes encoding MdsAB in *S. enterica*, and the MdsAB system can utilize both MdsC and TolC as OMPs to form a functional multidrug efflux pump [16]. This indicates that although the sequence similarity between TolC and MdsC is very low (15.7% in the sequence similarity and 6.1% sequence identity; only the sequence similarity is expressed hereafter), the mechanism by which they interact with MdsA to form MdsAB-mediated efflux systems is common.

Our research group has investigated the assembly of various tripartite efflux pumps, and proposed a cogwheel-like model for the interaction between MFPs (MacA, AcrA, HlyD, and MexA) and OMPs (TolC and OprM) in gram-negative bacteria [8], [14], [19]–[24]. This model proposes that the α-helical tip region of the MFP creates a cogwheel-to-cogwheel interaction with the aperture tip region of the OMP channel. Crystal structures combined with data from genetic studies have revealed that MacA exhibits a funnel-like hexameric assembly in their functional MacAB-TolC states and that the tip region of the α-barrel of MacA interacts with TolC [8], [20], [22]. We also observed that the α-barrel domain of OprM from the MexAB-OprM system of *Pseudomonas aeruginosa* resembled a 6-bladed cogwheel that is tightly linked to a complementary cogwheel structure in the MexA hexamer [23]. Our model has been recently supported by other studies. Reffay et al. showed that MexA and OprM do not interact laterally; instead, they form a functional complex by varying between two and six MexA molecules per OprM trimer, depending on the pH (7.5 to 5.5) [25]. In the case of the CusBAC copper efflux system, CusB, which is the MFP that binds to RND-type CusA, assumes a funnel-like hexameric assembly with CusA, providing further evidence for the hexamerization of MFP [26].

In this study, we investigated the functional role of the putative tip regions of the MdsA α-hairpin and the MdsC α-barrel of the *Salmonella*-specific MdsAB-MdsC efflux pump. By showing that one MFP can bind to two different OMP proteins using tip regions of them, we provided experimental evidence that this mode of interaction between MFPs and OMPs is part of an assembly mechanism in the formation of functional tripartite efflux pumps in gram-negative bacteria.

## Materials and Methods

### Strains and plasmids

Bacterial strains (all derivatives of *Salmonella enterica* serovar Typhimurium ATCC14028S) and plasmids used in this study are listed in Table 1. Bacterial strains were grown at 37°C in Luria-Bertani (LB) (1% tryptone, 0.5% yeast extract, and 0.5% NaCl) broth. Primers used are described in Table S1. The *mdsAB* genes were amplified from ATCC14028S genomic DNA by PCR, introducing an *Nde*I site at the 5′ end of *mdsA* and an *Xba*I site and the c-myc sequence at the 3′ end of *mdsB*. The PCR fragments were cloned into the *Nde*I and *Xba*I sites of pKAN6B under the control of a pBAD promoter to yield pMdsAB. To add a flag tag to the 3′ end of *mdsA*, the target regions were amplified and recombined using an overlap extension PCR method, resulting in pMdsAB2, which encodes MdsA-flag and MdsB-myc. Plasmids pMdsAB2-MdsA R135D, -MdsA L139D, and -MdsA S146D were constructed using the overlap extension PCR method. The resulting PCR fragments were digested with *Nde*I/*Sse*8387I and ligated into the same sites in pMdsAB2. PCR-amplified fragments encoding MdsB-myc and MdsC-hexahistidine were digested with *Sse*8387I and *Xba*I and ligated into the same sites in pMdsAB2 to produce pMdsABC2. Using the overlap extension PCR method, the resulting PCR DNA fragments, including MdsA R135D, MdsA L139D, and MdsA S146D, were digested with *Nde*I/*Sse*8387I and ligated into the same sites in pMdsABC2. To construct plasmids pMdsABC2-MdsC-G220A, -G433A, and -L441R, the overlap extension PCR-amplified fragments encoding MdsC-G220A, MdsC-G433A, and MdsC-L441R were digested with *Asc*I/*Xba*I, and then ligated into the same sites in pMdsABC2 to produce pMdsABC2-MdsC-G220A, MdsC-G433A, and MdsC-L441R, respectively. To construct partially truncated mutants of pMdsABC2, plasmids pMdsABC2-MdsC G220A, G433A, and L441R, were digested with *Hpa*I. After the excised fragments were discarded, the remaining fragments were ligated to construct pMdsC2, pMdsC2-G220A, pMdsC2-G433A, and pMdsC2-L441R.

**Table 1:** Bacterial strains and plasmids used in this study.

Strain or plasmid	Relevant characteristics	Reference
ATCC14028S	*Salmonella enterica* serovar Typhimurium (wild type)	[34]
*ΔacrAB*	ΔacrAB	This study
*ΔacrABmdsABC*	ΔacrABΔmdsABC::Cm^R^	This study
*ΔacrABmdsABCtolC*	ΔacrABΔmdsABC::Cm^R^ΔtolC::Tn^R^	This study
**Vectors**		
pKD3	rep_R6Kγ[p]_ Ap^R^ FRT Cm^R^ FRT	[27]
pCP20	rep_pSC101_^ts^AP^R^ Cm^R^ *cl857*λP_R_ *flp*	[27]
pKAN6B	p15A *ori*, Km^R^	[35]
pACYC184	p15A *ori*, Tn^R^, Cm^R^	[36]
**Plasmids**		
pMdsAB	*mdsAB* genes cloned into pKAN6B, Km^R^, expresses a c-terminal myc-tagged MdsB (MdsB-c-myc)	This study
pMdsAB2	Same as pMdsAB, but also expresses a c-terminal flag-tagged MdsA (MdsA-c-flag)	This study
pMdsAB2-MdsA-R135D	Same as pMdsAB2, but expresses an MdsA R135D mutant	This study
pMdsAB2-MdsA-L139D	Same as pMdsAB2, but expresses an MdsA L139D mutant	This study
pMdsAB2-MdsA-S146D	Same as pMdsAB2, but expresses an MdsA S146D mutant	This study
pMdsABC2	*mdsAB* genes cloned into pKAN6B, Km^R^, expresses MdsA-c-flag, MdsB-c-myc, and MdsC-c-hexahistidine	This study
pMdsABC2-MdsA-R135D	Same as pMdsABC2, but expresses an MdsA R135D mutant	This study
pMdsABC2-MdsA-L139D	Same as pMdsABC2, but expresses an MdsA L139D mutant	This study
pMdsABC2-MdsA-S146D	Same as pMdsABC2, but expresses an MdsA S146D mutant	This study
pMdsABC2-MdsC-G220A	Same as pMdsABC2, but expresses an MdsC G220A mutant	This study
pMdsABC2-MdsC-G433A	Same as pMdsABC2, but expresses an MdsC G433A mutant	This study
pMdsABC2-MdsC-L441R	Same as pMdsABC2, but expresses an MdsC L441R mutant	This study
pMdsC2	Same as pMdsABC2, but with truncated parts of MdsA and MdsB	This study
pMdsC2-G220A	Same as pMdsC2, but expresses an MdsC G220A mutant	This study
pMdsC2-G433A	Same as pMdsC2, but expresses an MdsC G433A mutant	This study
pMdsC2-L441R	Same as pMdsC2, but expresses an MdsC L441R mutant	This study

To construct *ΔmdsABC*, *ΔacrAB*, and *ΔtolC* strains, gene disruption was performed as previously described [27]. The chloramphenicol resistance (*cat*) gene for the *mdsABC* gene deletion, the *cat* gene with Flp recognition sites for the *acrAB* gene deletion, and the tetracycline resistance (*Tn*) gene for the *tolC* gene deletion were amplified by PCR with the oligonucleotide primers listed in Table S1. The resulting PCR products were transformed into the ATCC14028S strain harboring plasmid pKD46, which expresses the Red recombinase. The chromosomal structure of the mutated loci was verified by PCR. In the case of the *acrAB* deletion, the *cat* gene was removed by using plasmid pCP20 as described above.

### Measurement of the MIC of toxic compounds

The measurement of MIC has been previously described [19]. Overnight cultures grown in LB medium with the appropriate antibiotics (100 µg of kanamycin, 5 µg of chloramphenicol, and 5 µg of tetracycline per mL) were diluted 1∶100 in the same medium. At an optical density at 600 nm (OD_600_) of 0.1, the expression of proteins was induced by adding 0.1% arabinose to the cultures and incubating for 100 min. Approximately 10^4^ induced cells were added to the same medium containing 0.1% arabinose and crystal violet, methylene blue, acriflavine, rhodamine 6G, and vancomycin in increasing concentrations. The cultures were grown for an additional 16 to 18 h, and the lowest concentrations of crystal violet, methylene blue, acriflavine, rhodamine 6G, and vancomycin that completely inhibited growth were designated as the MICs.

### *In vivo* cross-linking assay

The procedure for *in vivo* cross-linking using Dithiobis (succinimidyl propionate) (DSP) has been described previously [28]. ATCC14028S*ΔacrABΔmdsABC::Cm5ΔtolC::Tn10* cells carrying an empty vector, pMdsABC2, or its derivatives (MdsA-R135D, MdsA-L139D, MdsA-S146D, MdsC-G220A, MdsC-G433A, and MdsC-L441R) were grown in LB medium with the appropriate antibiotics to OD_600_ = 0.8, and then used for cross-linking experiments. Anti-his and anti-flag monoclonal antibodies were used to detect MdsC with a c-terminal hexahistidine tag and MdsA with a c-terminal flag tag, respectively.

## Results

### Sequence analysis of the putative MdsA α-hairpin and MdsC α-barrel tip regions

**Figure 1:**
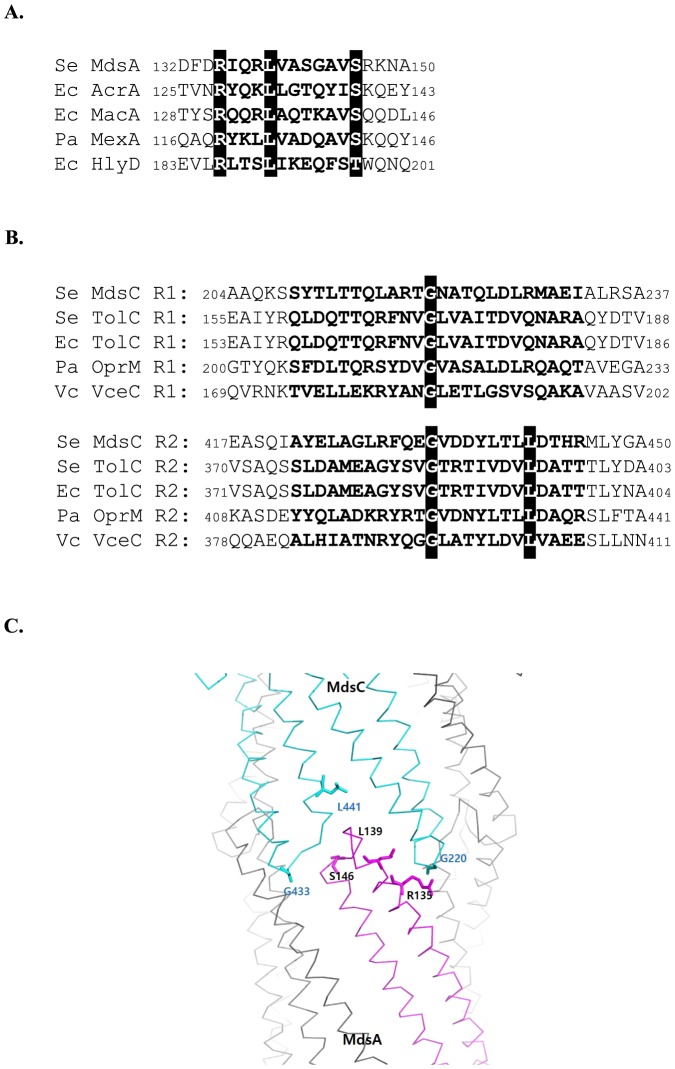
Sequence comparisons of the putative tip regions of MdsA and MdsC. (A) Sequence alignment of the RLS motifs from three distinct adapter proteins. The corresponding heptad positions are marked in bold. The three conserved residues are highlighted in the black box. (B) Conserved amino acid residues in repeat 1 and repeat 2 in the aperture tip region of OMP. Repeat sequences in the tip regions are shown in bold, and conserved residues are shown in black boxes. Se, *Salmonella enterica*; Ec, *Escherichia coli*; Pa, *Pseudomonas aeruginosa*; Vc, *Vibrio cholerae*. (C) A modeled complex structure of the α-hairpin tip regions of MdsA and MdsC structure, based on the adaptor bridging model [23]. The residues mentioned in the text are shown in the stick representations.

Since the MdsA protein can associate with either MdsC or TolC to form a functional pump, we hypothesized that MdsA has a common structural motif allowing it to bind to both MdsC and TolC. We hypothesized that this structural motif could be the conserved RxxxLxxxxxxS/T (RLS) motif located in the α-hairpin tip regions of MFPs such as MacA, AcrA, and HlyD that creates a binding interface for the TolC α-barrel tip [20]–[22], [ 24]. The amino acid sequence alignment shows that the RLS motif in the α-helical tip region of MdsA is strongly conserved even though MdsA does not share high sequence similarity with other MFPs (15%, 18.7%, and 14% with MacA, AcrA, and HlyD, respectively) (Figure 1A). Previous studies showed that the guanidium group of the first residue in the RLS motif forms a hydrogen bond with the backbone carbonyl group of the α-barrel tip region in the OMP [21]–[23]. The leucine residue of the RLS motif in MFPs is the most conserved amino acid residue, which mediates strong tip-to-tip interactions between MFPs and OMPs. However, the last serine residue of this motif often has sequence flexibility.

Considering that the α-barrel region of TolC binds to AcrA or MacA to form an efflux pump [15], [19], [20]–[22], we assumed that the aperture tip region of the oligomeric, assembled MdsC protein would also be crucial for interaction with MdsA (Figure 1B). The MdsC protein has extremely low sequence similarity to TolC (15.6%); however, it shares approximately 50% sequence similarity with *P. aeruginosa* OprM. Nonetheless, according to the structured-based sequence alignment, TolC, OprM, and MdsC share similar functional domains (Figure 1B). Specifically, Gly220 of repeat 1 and Gly433 of repeat 2 in MdsC are particularly well conserved across other OMPs in gram-negative bacteria. Based on previous structural studies on MexA and OprM, the glycine located in the Thr-Gly-Asn residues of repeat 1 and the Glu-Gly-Val residues of repeat 2 are expected to be involved in the direct interaction with Leu139 of MdsA [23]. In addition, Leu395 in TolC (Leu373 excluding the signal peptide sequence; 1–22) of *E. coli*, or the equivalent residue in the TolCV1 homolog (Leu369 excluding the putative signal peptide sequence) of *Vibrio vulnificus*, has been known to affect the functionality of tripartite efflux pumps [15], [19], [25]; these conserved residues also exists in MdsC (at Leu441). Figure 1C indicates the location of six important residues in the tip regions of MdsA and MdsC that have been predicted to be involved in an interaction between MdsA and MdsC based on the adaptor bridging model [23]. Taken together, these results suggest that the putative tip regions of MdsA and MdsC share a structural motif involved in its assembly with other tripartite efflux pumps despite the very low sequence similarities shared with other MFPs and OMPs.

### The conserved residues in the MdsA α-hairpin tip region are functionally and physically significant

**Figure 2:**
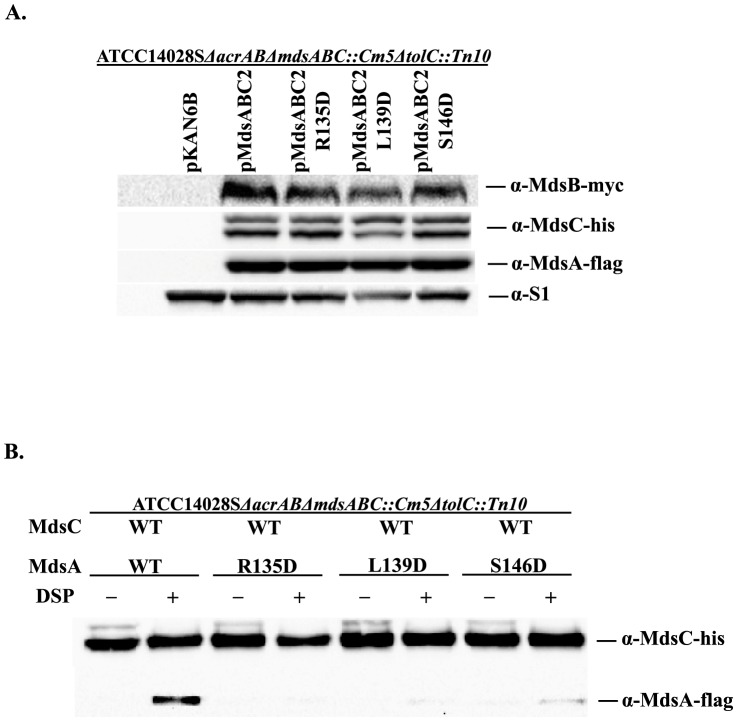
Physical interaction between MdsA or MdsA variants and MdsC *in vivo*. (A) Protein expression of Flag-tagged MdsA and MdsA variants (MdsA-R135D, MdsA-L139D, and MdsA-S146D), MdsB-Myc, and hexahistidine-tagged MdsC was detected by western blotting. (B) The *in vivo* interaction between MdsA and MdsC was analyzed by using the chemical-crosslinker DSP. *S. enterica* ATCC14028S*ΔacrABΔmdsABC::Cm5ΔtolC::Tn10* cells coexpressing c-flag-tagged wild-type (WT) or mutant MdsA (R135D, L139D, and S146D), c-myc-tagged MdsB, hexahistidine-tagged MdsC were tested. All cultures were treated with (+) or without (−) DSP. Affinity-purified MdsC and crosslinked MdsA and MdsA variants were separated by SDS-PAGE and immunoblotted using monoclonal anti-His-tag and anti-Flag-tag antibodies.

To examine whether the MdsA α-hairpin region is capable of interacting with the α-barrel tip region of the cognate OMP, MdsC, we mutated three conserved residues (Arg135, Leu139, and Ser146) to Asp and tested the ability of these mutant proteins to form a functional drug efflux pump. To measure the function of the MdsAB-MdsC efflux pump, it was necessary to eliminate the genes encoding the major multidrug resistance pump *acrAB* as well as *mdsABC* [16]. Because MdsAB proteins can utilize TolC as an OMP, we also deleted the chromosomal copy of *tolC* in *S. enterica* (ATCC14028S *ΔacrABΔmdsABC::Cm5ΔtolC::Tn10*). As shown in Table 2, all of these changes (R135D, L139D, and S146D) in the putative α-hairpin tip region of MdsA appeared to result in a loss of MdsAB-MdsC pump function because *S. enterica* cells expressing these MdsC mutants exhibited the same degree of susceptibility to several known substrates (crystal violet, methylene blue, acriflavine, and rhodamine 6G) as those harboring an empty vector. Western blot analysis of the MdsA proteins in these strains revealed that these mutations did not affect the steady-state levels of MdsA, indicating that the loss of efflux function was not related to expression levels or the stability of the mutant MdsA proteins (Figure 2A). These results indicate that the conserved residues, which are located in the tip region of MdsA, play an important role in the function of the MdsAB-MdsC pump *in vivo*.

**Table 2:** The *in vivo* effect of mutations in the conserved region of MdsA^a^ in the presence of the wild-type MdsB and MdsC and in the absence of TolC.

Background	ATCC14028S*ΔacrABΔmdsABC::Cm5ΔtolC::Tn10*			
MdsA protein	MIC (µg/mL)^b^			
	Crystal violet	Methylene blue	Acriflavine	Rhodamine 6G
None^c^	4	32	16	4
MdsA-WT	16	256	32	64
MdsA-R135D	4	32	16	4
MdsA-L139D	4	32	16	4
MdsA-S146D	4	32	16	4

a The *in vivo* effect of mutations in the conserved region of MdsA was determined by measuring the resistance of the ATCC14028S*ΔacrABΔmdsABC::Cm5ΔtolC::Tn10* strain to several substrates. MdsA and its variants were expressed from pMdsABC2, pMdsABC2 MdsA-R135D, pMdsABC2 MdsA-L139D, and pMdsABC2 MdsA-S146D.

b All MIC measurements were done in triplicate. The concentrations of crystal violet and acriflavine used to determine the MICs were 0, 1, 2, 4, 8, 16, and 32 µg/mL. The concentrations of rhodamine 6G and methylene blue used to determine the MICs were 0, 4, 8, 16, 32, 64, 128, and 256 µg/mL.

c None, strain carrying the empty vector pKAN6B instead of a pMdsABC2 variant.

Previous reports showed that single amino acid substitutions in the RLS motifs of other MFPs, including AcrA, MacA, and HlyD, resulted in a loss of TolC binding [15], [20]–[22], [24]. Based on these findings, we performed *in vivo* chemical cross-linking to further examine the physical interactions between the MdsA α-hairpin tip region and MdsC. Exponential cultures of the same strains used for MIC measurements were crosslinked with the chemical crosslinker DSP. Hexahistidine-tagged MdsC and cross-linked, flag-tagged MdsA were isolated by using a Ni-NTA resin column. As shown in Figure 2B, the reduced resistance of the MdsAB-MdsC pump caused by the mutations in the MdsA RLS motif was associated with a loss of the physical interaction between MdsA and MdsC. The crosslinking of MdsA-S146D to MdsC was only 16% of that of wild-type MdsA and MdsC, whereas no crosslinking of MdsA-R135D or MdsA-L139D to MdsC was detected. These results indicated that an amino acid substitution in the RLS motif either partially (S146D) or completely (R135D and L139D) abolished the binding of MdsA to MdsC *in vivo* and consequently lead to a loss of MdsAB-MdsC pump function.

Since the MdsAB system relies on the presence of either MdsC or TolC for drug resistance, we hypothesized that the invariant residues of MdsA are also important for the interaction with TolC in the MdsAB-TolC system. By determining the MIC of several substances for a set of isogenic *S. enterica* strains deleted for *acrAB* and *mdsABC* (ATCC14028S*ΔacrABΔmdsABC*) that express wild-type *mdsAB*, *mdsAB*-R135D, *mdsAB*-L139D, or *mdsAB*-S146D from plasmid pMdsAB2 or its derivatives (Figure S1), we examined the effect of mutations in the RLS motif of MdsA on the efflux function of the MdsAB-TolC pump (Table 3). Both the R135D and L139D mutations resulted in markedly decreased MIC resistance, whereas the S146D mutation did not affect resistance to the substances tested. It has been shown that the third serine residue of the RLS motif is flexible [22] and, in other MFPs, it is often a different amino acid [24]. In some cases, introduction of a mutation at the third residue of the RLS motif in MFPs had a less significant effect on efflux pump function than mutation of the other residues (Arg and Leu) [23], [24]. Taken together, these results show that the conserved residues of the MdsA α-hairpin tip region are functionally significant for the interaction with both MdsC and TolC in the MdsAB-MdsC and MdsAB-TolC systems, respectively.

**Table 3:** The *in vivo* effect of mutations in the conserved region of MdsA^a^ in the presence of wild-type MdsB and TolC without MdsC.

Background	ATCC14028S*ΔacrABΔmdsABC::Cm5*			
MdsA protein	MIC (µg/mL)^b^			
	Crystal violet	Methylene blue	Acriflavine	Rhodamine 6G
None^c^	4	32	16	4
MdsA-WT	16	256	32	64
MdsA-R135D	8	64	16	32
MdsA-L139D	4	32	16	8
MdsA-S146D	16	256	32	64

a The *in vivo* effect of each mutation in the conserved region of MdsA was determined by measuring the resistance of the ATCC14028S*ΔacrABΔmdsABC::Cm5* strain to several substrates. MdsA and its variants were expressed from pMdsAB2, pMdsAB2 MdsA-R135D, pMdsAB2 MdsA-L139D, and pMdsAB2 MdsA-S146D.

b All MIC measurements were done in triplicate. The concentrations of crystal violet and acriflavine used to determine the MICs were 0, 1, 2, 4, 8, 16, and 32 µg/mL. The concentrations of rhodamine 6G and methylene blue used to determine the MICs were 0, 4, 8, 16, 32, 64, 128, and 256 µg/mL.

c None, strain carrying the empty vector pKAN6B instead of an pMdsAB2 variant.

### The MdsC aperture tip region is responsible for its interaction with MdsA

Previous studies showed that the TolC aperture tip region is functionally related to the AcrA α-hairpin tip region using genetic complementation and *in vivo/in vitro* binding assays [15], [19]. To determine whether the aperture tip region of MdsC is functionally related to the assembly of the MdsAB-MdsC pump, we performed site-directed mutagenesis of the glycine residues (Gly220 and Gly433) in two conserved motifs (the Thr-Gly-Asn motif of repeat 1 and the Glu-Gly-Val motif of repeat 2) located in the putative tip region of MdsC. In addition, the *E. coli* TolC L395R mutation, which was previously identified by genetic complementation, is also known to disrupt the interaction between AcrA and TolC [22]. In particular, the leucine residue would be located at the cogwheel structure in the putative open conformation of MdsC or TolC. To see the functional importance of the leucine, we substituted the corresponding position (Leu441) of MdsC with Arg and measured the sensitivity of the mutant to various toxic compounds (Table 4). Substitution of the conserved glycine residues in repeat 1 and repeat 2 with alanine resulted in a loss of MdsAB-MdsC pump function and analogous result was obtained for the L441R mutation, confirming the functional importance of the residues.

**Table 4:** The *in vivo* effect of mutations at the aperture tip region of MdsC^a^ in the presence of wild type MdsA and MdsB^a^.

Background	ATCC14028S*ΔacrABΔmdsABC::Cm5ΔtolC::Tn10*				
MdsC protein	MIC (µg/mL)^b^				
	Crystal violet	Methylene blue	Acriflavine	Rhodamine 6G	Vancomycin^c^
None^d^	4	32	16	4	800
MdsC-WT	16	256	32	64	700
MdsC-G220A	4	32	16	4	500
MdsC-G433A	4	32	16	4	600
MdsC-L441R	4	32	16	4	700

a The *in vivo* effect of mutations in the aperture tip region of MdsC was determined by measuring the resistance of the ATCC14028S*ΔacrABΔmdsABC::Cm5ΔtolC::Tn10* strain to several substrates. MdsC and its variants were expressed from pMdsABC2, pMdsABC2 MdsC-G220A, pMdsABC2 MdsC-G433A, pMdsABC2 MdsC-L441R.

b All MIC measurements were done in triplicate. The concentrations of crystal violet and acriflavine used to determine the MICs were 0, 1, 2, 4, 8, 16, and 32 µg/mL. The concentrations of rhodamine 6G and methylene blue used to determine the MICs were 0, 4, 8, 16, 32, 64, 128, and 256 µg/mL.

c The vancomycin concentrations used to determine the MICs were 0, 300, 400, 500, 600, 700, and 800 µg/mL.

d None, strain carrying the empty vector pKAN6B instead of a pMdsABC2 variant.

When the channel aperture was permanently altered to an open state by disrupting the hydrogen bonding network between the inner helices with introduction of the Y362F and R367E mutations in *E. coli* TolC, *E. coli* became sensitive to this large vancomycin by allowing the antibiotics to pass through a constitutively open channel [29], [30]. To examine whether the tested residues MdsC is involved in keeping the MdsC channel closed, leading to abolishing the function of the MdsAB-MdsC efflux pump, we tested the vancomycin sensitivity of *S. enterica* strains that expressed wild-type or the mutant MdsC proteins. As shown in Table 4, the mutations did not significantly affect the susceptibility of *S. enterica* cells to vancomycin, indicating that these mutations would affect the binding to MdsA (or MdsB) for function of the pump without causing a leaky channel or destruction of the MdsC closing mechanism.

**Figure 3:**
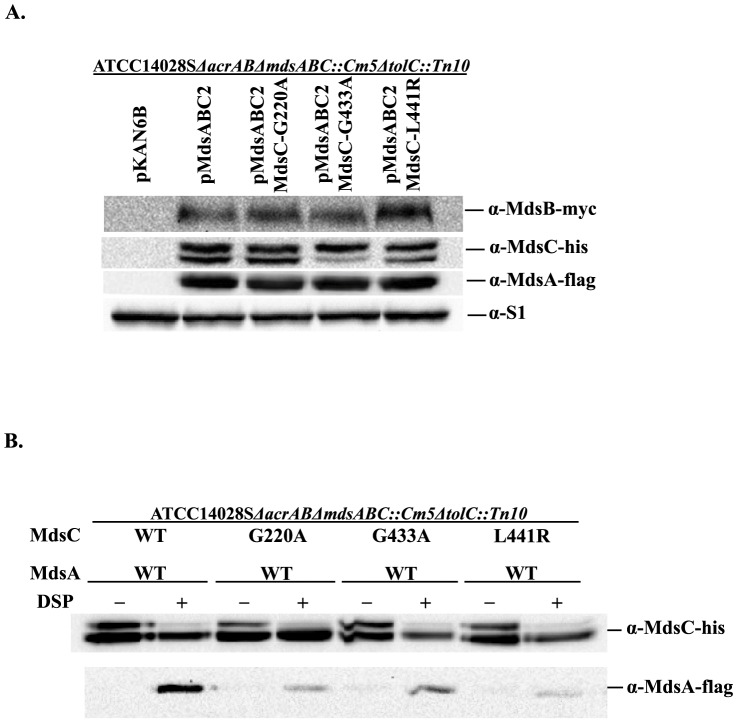
Interaction between MdsC or MdsC variants and MdsA *in vivo*. (A) Protein expression of hexahistidine-tagged MdsC and MdsC variants (MdsC-G220A, MdsC-G433A, and MdsC-L441R), MdsA-Flag and MdsB-Myc was detected by western blotting. (B) The *in vivo* interaction between MdsA and MdsC or MdsC variants was detected by using the chemical-crosslinker DSP. *S. enterica* ATCC14028S*ΔacrABΔmdsABC::Cm5ΔtolC::Tn10* cells coexpressing c-Flag-tagged MdsA, c-Myc-tagged MdsB, and hexahistidine-tagged wild-type (WT) or mutant MdsC (G220A, G433A, and L441R) are shown. All cultures were treated with (+) or without (−) DSP. Affinity-purified MdsC or MdsC variants and crosslinked MdsA were separated by SDS-PAGE and immunoblotted using monoclonal anti-His-tag and anti-Flag-tag antibodies.

Once again, we employed *in vivo* DSP-mediated crosslinking to determine if a loss of efflux function caused by mutations in the MdsC aperture tip region is associated with a defect in MdsA binding. As shown in Figure 3B, compared to the wild-type MdsC protein, 20%, 39%, and 16% of the MdsA protein crosslinked to the MdsC variants G220A, G433A, and L441R, respectively. These results implied that the impaired function of efflux pumps caused by the mutations in the MdsC aperture tip region stems from a loss of interaction with MdsA. Based on these results, we further examined whether the results of the vancomycin susceptibility test are affected by the inability of the MdsC mutants to interact with MdsA and form the MdsAB-MdsC complex. To exclude the effect of interaction with MdsAB on the ability of MdsC mutants to allow the diffusion of vancomycin, we used *S. enterica* strains that did not express MdsA and MdsB for these experiments. The results showed that the expression of these MdsC mutants did not significantly affect the resistance of *S. enterica* cells to vancomycin (Table 5), confirming that the mutations in the MdsC aperture tip region did not disturb the maintenance of a closed aperture in MdsC as was previously shown for TolC homologs [31].

**Table 5:** The *in vivo* effect of mutations at the aperture tip region of MdsC in the absence of functional MdsA, MdsB, and TolC^a^.

Background	ATCC14028S*ΔacrABΔmdsABC::Cm5ΔtolC::Tn10*				
MdsC protein having no functional MdsA-MdsB	MIC (µg/mL)^b^				
	Crystal violet	Methylene blue	Acriflavine	Rhodamine 6G	Vancomycin
MdsC-WT	4	32	16	4	800
MdsC-G220A	4	32	16	4	800
MdsC-G433A	4	32	16	4	800
MdsC-L441R	4	32	16	4	800

a The *in vivo* effect of mutations in the aperture tip region of MdsC when wild-type MdsA and MdsB were disrupted was determined by measuring the resistance of the ATCC14028S*ΔacrABΔmdsABC::Cm5ΔtolC::Tn10* strain to several substrates. MdsC and its variants were expressed from pMdsC2, pMdsC2-G220A, pMdsC2-G433A, and pMdsC2-L441R.

b All MIC measurements were done in triplicate. The concentrations of crystal violet and acriflavine used to determine the MICs were 0, 1, 2, 4, 8, 16, and 32 µg/mL. The concentrations of rhodamine 6G and methylene blue used to determine the MICs were 0, 4, 8, 16, 32, 64, 128, and 256 µg/mL. The vancomycin concentrations used to determine the MICs were 0, 300, 400, 500, 600, 700, and 800 µg/mL.

## Discussion

Functional genetics and structural analyses have shown that MFPs such as AcrA and MacA function as funnel-like hexamers and interact with OMPs in a tip-to-tip manner [19], [22], [23]. Although the overall structure of the *Salmonella*-specific MdsAB-MdsC pump has not yet been determined, we showed that the previously determined assembly model can be applied to the MdsAB-mediated system. Sequence comparison showed that the putative tip region of MdsA has the evolutionarily well-conserved RLS motif (Arg135, Leu139, and Ser146) even though the MdsA amino acid sequence shares very limited similarity with other MFPs. Previous studies showed that these residues mediate tip-to-tip interactions between the MFP and OMP [23]. Consistent with this observation, a single mutation in the conserved residues of the aperture tip region of MFPs, including AcrA, MacA, and HlyD, resulted in reduced efflux activity [19], [20], [24]. These MFPs commonly utilize TolC as an OMP for their efflux systems, such as AcrAB-TolC, MacAB-TolC, and HlyDB-TolC. Likewise, MdsA can form a functional complex with TolC instead of MdsC. In this study, we obtained analogous results from a mutational analysis of the RLS motif in MdsA, indicating that the RLS motif in MdsA plays an important functional role by physically interacting with its OMPs, MdsC and TolC.

This tip-to-tip interaction between MdsA and MdsC was further supported by our analyses of MdsC variants containing mutations at the conserved glycine residues in the putative tip region of MdsC (Table 4 and Figure 3). These conserved glycine residues are present in an invariant motif, Val/Thr-Gly-Val (V/TGV), at the α-barrel tip region of OMPs such as TolC, OprM, and CusC. This V/TGV motif was shown to make a characteristic sharp bend, which results in the Val/Thr and Val residues facing in the same direction [23]. In particular, the second leucine residue of the TolC RLS motif makes a strong leucine zipper-like interaction with the OMP V/TGV motif in both repeats 1 and 2 [23]. Thus, the putative aperture tip region of MdsC may also form a structural motif similar to that of *E. coli* TolC, which plays a key role in its interaction with MFPs.

The mode of interaction between various the MFPs and OMPs that we have investigated was also recently found in the *E. coli* CusCBA system, which extrudes metal ions such as Cu and Ag from either the cytoplasm or the periplasm to the extracellular environment [26]. The co-crystal structure of the CusBA efflux complex has shown that the trimeric inner membrane transporter CusA interacts with six molecules of the MFP CusB. The side-by-side packing arrangement of six CusB protomers creates a funnel-like structure to form a channel for the active extrusion of metal ions [26], and these structural data fit our adapter bridging model of tripartite efflux pumps in gram-negative bacteria. This model can also explain why second-site mutations that can suppress loss-of-function mutations in AcrB β-hairpin loops are found in the β-barrel domain of AcrA [32]. We think that the simplest interpretation of these results is a direct interaction between the AcrA β-barrel domain and AcrB β-hairpin loops. However, the current assembly model of AcrAB-TolC based on the tip-to-tip AcrB-TolC interaction and side-by-side AcrA-TolC interaction proposes that AcrB β-hairpin loops act as TolC-docking domains [33]. We believe that these tip-to-tip AcrB-TolC and the side-by-side AcrA-TolC interactions might be important in a certain step during the formation of the functional complex.

The experimental results from our functional analysis of the putative tip regions of MdsA and MdsC are consistent with those of the AcrAB-TolC, MacAB-TolC, MexAB-OprM, and HlyDB-TolC complexes, indicating the existence of a common mode of interaction between various MFPs and OMPs. Although further studies are required to determine the exact assembly mechanism underlying the formation of a functional MdsAB-mediated pump, the results of this study contribute to our understanding of the general assembly mechanism for tripartite efflux pumps.

## Supporting Information

Figure S1 **Protein expression of MdsA or MdsA variants and MdsB.** Protein expression of Flag-tagged MdsA and MdsA variants (MdsA-R135D, MdsA-L139D, and MdsA-S146D) and Myc-tagged MdsB was detected by western blotting.
(EPS)

Table S1 **Primers used in this study.**
(DOC)
