# Supplementary material for: Interaction Mediated by the Putative Tip Regions of MdsA and MdsC in the Formation of a Salmonella-Specific Tripartite Efflux Pump
Source: PLoS One. 2014 Jun 24;9(6):e100881. doi: 10.1371/journal.pone.0100881 (PMC4069162; doi:10.1371/journal.pone.0100881)
Supplement: Table S1 — Primers used in this study. (DOC) [file pone.0100881.s002.doc]

**Table S1. Primers used in this study.**

| **Primer** | **Sequence (5ʹ to 3ʹ)** |
| --- | --- |
| **For gene deletion** |  |
| AcrA-H1P1 | TTGAAATCGGACACTCGAGGTTTACATATGAACAAAAACGTGTAGGCTGGAGCTGCTTC |
| AcrB-H2P2 | CGGCTAAAGCGGCGGCGTACCACCACGAAGAAGACCGGTATATGAATATCCTCCTTA |
| TolC-H1P1 | TCAGCGCTAAATACTGCTTCACAACAAGGAATGCAAATGAGCACCTGAAGTCAGCCCCA |
| TolC-H2P2 | TTGCCGTTATTGCTGTTGGCGCGAGCGGCGGTCGGCTGTGAGTGGTGAATCCGTTAGCG |
| MdsA-H1P1 | TGCGGAATGGCCGACACGCTGCGGTAAAGAGGGAAACGACGTGTAGGCTGGAGCTGCTTC |
| MdsC-H2P2 | TAGCGCGCGCGCCGCACTGGCGACGCGGGCGCTATTTGGGCATATGAATATCCTCCTTAG |
| **For gene cloning** |  |
| MdsA-F_*Nde*I | CGCATATGCGTAGAACATTCAAAATTATGTTGATAGC |
| MdsB-R_*Xba*I | TATCTAGATTATGCTTGCTGATCATGCG |
| MdsB-F_*Sse*8387I | GGCCTGCAGGGTGAGTTTTATCGTCAG |
| MdsB-R_*Sse*8387I | AACTCACCCTGCAGGCCCGCCAGGAATG |
| MdsA-F_Flag | GCCAAGACTACAAGGACGACGACGACAAATGAAATTCACCCACTTTTTCATTGCACGCCCCA |
| MdsA-R_Flag | GAATTTCATTTGTCGTCGTCGTCCTTGTAGTCTTGGCGCACCGCCTTCGGCGCACTGTCG |
| MdsB-R_HpaI | GCCGTTAACCGACAGACC |
| MdsB-F_Myc | GCAGAACAGAAACTGATCTCTGAAGAAGACCTGTAATGATGGGAATGGCATAATGAAGATCAC |
| MdsB-R_Myc | TACAGGTCTTCTTCAGAGATCAGTTTCTGTTCTGCTTGCTGATCATGCGAATCAACGCGGTTCTC |
| MdsB-R_Myc_XbaI | CGTCTAGATTACAGGTCTTCTTCAGAGATCAGTTTCTGTTCTGCTTGCTGATCATGCGAATCAAC |
| MdsC-R_His_XbaI | CGTCTAGACTAATGATGATGATGATGATGACCTTGCTTTTTCTCACTGTATTCCCGCCA |
| MdsA-F_R135D | CAGGCGGATTTCGATGATATTCAACGACTGGTCG |
| MdsA-R_R135D | GACCAGTCGTTGAATATCATCGAAATCCGCCTGC |
| MdsA-F_L139D | GATCGCATTCAACGAGATGTCGCCAGCGGCGCCGT |
| MdsA-R_L139D | ACGGCGCCGCTGGCGACATCTCGTTGAATGCGATCG |
| MdsA-F_S146D | CCAGCGGCGCCGTAGATCGTAAAAACGCTGACG |
| MdsA-R_S146D | AGCGTTTTTACGATCTACGGCGCCGCTGGCGAC |
| MdsA-R_ScaI | GCAGTACTCCCTGTGTAACGACGCGGAA |
| MdsC-F_G220A | CTTGCCCGGACAGCGAACGCCACACAGCTCG |
| MdsC-R_G220A | TGTGGCGTTCGCTGTCCGGGCAAGCTGGGTC |
| MdsC-F_G433A | GTTTTCAGGAAGCGGTCGATGACTACCTTAC |
| MdsC-R_G433A | TAGTCATCGACCGCTTCCTGAAAACGGAGTC |
| MdsC-F_L441R | CTACCTTACGCTGCGTGATACCCATCGTATGCT |
| MdsC-R_L441R | GCATACGATGGGTATCACGCAGCGTAAGGTAG |
| MdsC-F_AscI | GTTTCTGGCGCGCCGGATG |
